# *SERPING1* polymorphisms in polypoidal choroidal vasculopathy

**Published:** 2010-02-16

**Authors:** Meng Li, Feng Wen, Chengguo Zuo, Xiongze Zhang, Hui Chen, Shizhou Huang, Guangwei Luo

**Affiliations:** State Key Laboratory of Ophthalmology, Zhongshan Ophthalmic Center, Sun Yat-sen University, Guangzhou, China

## Abstract

**Purpose:**

To investigate whether common genetic variants in the complement component 1 inhibitor gene (serpin peptidase inhibitor, clade G, member 1, *SERPING1*) are associated with polypoidal choroidal vasculopathy (PCV) in a Chinese Han population.

**Methods:**

DNA samples were obtained from 118 PCV patients and 115 healthy subjects. Data derived from the HapMap project were used to select tag single nucleotide polymorphisms (SNPs) across the extended *SERPING1* region. A previously reported age-related macular degeneration-related risk factor (rs2511989) was forcibly included. Genotyping of each tag SNP was performed by PCR restriction fragment length polymorphism and direct DNA sequencing techniques.

**Results:**

Four SNPs for *SERPING1*, rs2509897, rs1005510, rs11603020, and rs2511989, were chosen as tag SNPs. None of these tag SNPs were associated with PCV, according to the single-SNP association test (p=0.41–0.83). Evaluation of common haplotypes across *SERPING1* did not reveal any association with PCV (p=0.49–0.82).

**Conclusions:**

We found no evidence to support the role of any common *SERPING1* variants, including the rs2511989 variant, in the susceptibility to PCV in a Chinese Han population.

## Introduction

Polypoidal choroidal vasculopathy (PCV) is a hemorrhagic and exudative macular disease that shows distinct forms of choroidal vascular abnormalities, including abnormal choroidal vascular networks and polypoidal lesions at their borders [1-3]. PCV has a particularly high incidence among the exudative age-related macular degeneration (AMD) in Asian populations, accounting for 24.5% of exudative AMD in the Chinese population [4] and 54.7% in the Japanese population [5] compared with only 8%–13% in Caucasians [3].

PCV shares many similarities with neovascular AMD, including demography [4,5], pathology [6-8], and manifestation [4,5]. Due to the similarities between PCV and neovascular AMD, most genetic risk factors for PCV were found based on previous genetic studies for AMD patients. Several genetic risk factors for AMD have been established, including the complement factor H (*CFH*) gene on chromosome 1q32 [9-13] and two tightly linked genes, namely age-related maculopathy susceptibility 2 (*ARMS2*) and high-temperature requirement factor A1 (*HTRA1*) on chromosome 10q26 [14-16]. Several recent studies have shown a strong association between PCV and these variants [17-22].

Since the discovery of the association between AMD and variants in *CFH*, several other genes regulating or involved in the alternative pathway of complement activation have been found to show a strong association with AMD, including those coding for complement component 3 (*C3*) [23], complement factor B (*CFB*), and complement factor 2 (*C2*) [24]. The complement factor 1 (*C1*) inhibitor is encoded by the serpin peptidase inhibitor, clade G, member 1 *(SERPING1)* gene (GenBank NM_000062) and is a member of a large family of serine proteases. The protein encoded by *SERPING1* is a key regulator of the classical pathway for complement activation. It has been reported to downregulate the alternative pathway in vitro by binding to *C3b* and inhibiting the binding of *CFB* to *C3b* [25]. Recently, Ennis et al. reported a protective effect on AMD for the minor allele of rs2511989 within intron 6 of *SERPING1* [26]. However, the association was not replicated in another study conducted by Park et al. [27].

Considering the similarities between PCV and neovascular AMD and the strong association between PCV and *CFH*, we hypothesized that polymorphisms in the *SERPING1* gene, another regulator of the complement system, might play a role in the development of PCV. In this study, we genotyped four tag single nucleotide polymorphisms (SNPs) that are highly representative of the common genetic variations in the *SERPING1* region and analyzed the associations between these variants and PCV in a Chinese Han population.

## Methods

### Study participants

The study protocol was approved by the institutional review board at the Zhongshan Ophthalmic Center of Sun Yat-sen University, Guangzhou, China, and performed in accordance with the Declaration of Helsinki. Informed consent was obtained from all subjects before participation in this study. All cases and controls included in this study were Chinese Han individuals recruited from the Zhongshan Ophthalmic Center.

**Figure 1:**
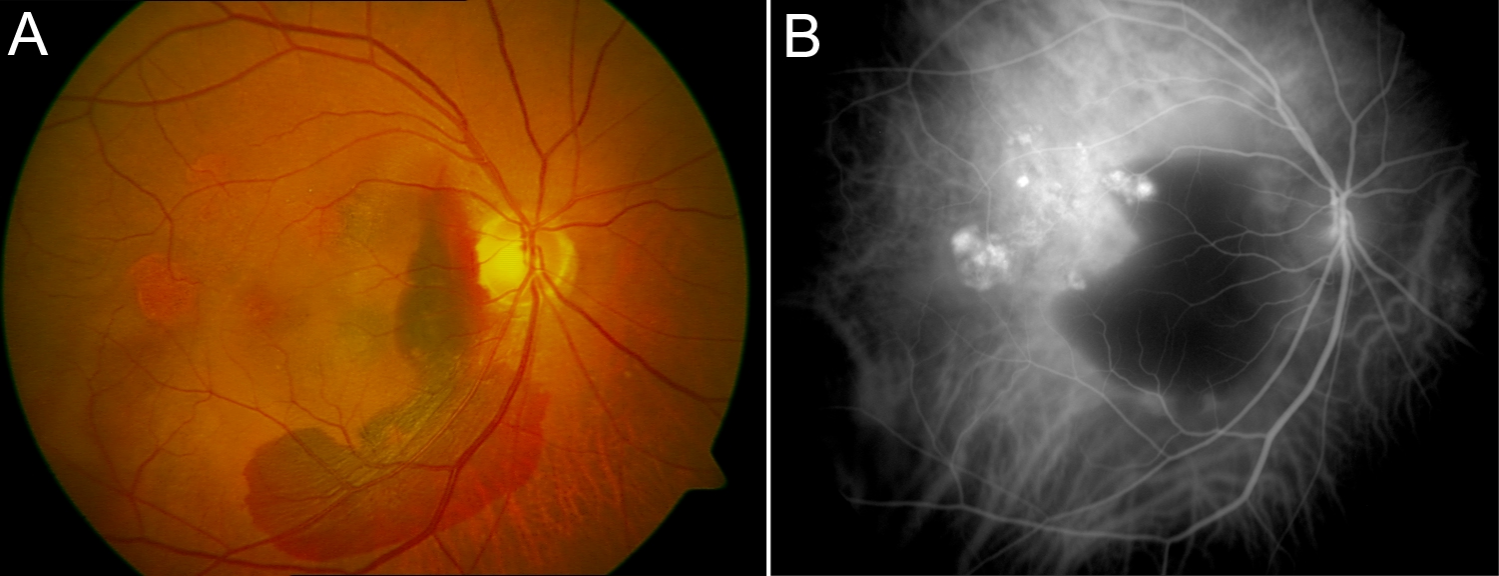
Clinical photos of polypoidal choroidal vasculopathy (PCV). **A**: Color fundus photograph of a patient with PCV. Several orange lesions are visible in the macula. Hemorrhage is visible between the orange lesions and the optic disk. **B**: The diagnosis of PCV was confirmed with indocyanine green angiography (ICGA). Abnormal choroidal vascular networks and characteristic polypoidal lesions are visible in the macula, corresponding to the orange lesion in the color fundus photograph.

All PCV patients underwent ophthalmic examinations, including visual acuity measurements, slit-lamp biomicroscopy, ophthalmoscope, color fundus photographs, fluorescein angiography, and indocyanine green angiography (ICGA). The diagnosis of PCV was based on identification of polypoidal choroidal vascular dilations with or without branching inner choroidal vessels on ICGA. Thus, all PCV patients enrolled in this study met the criteria of definitive cases of PCV, as proposed by the Japanese Study Group of Polypoidal Choroidal Vasculopathy [28]. Definitive cases of PCV must satisfy at least one of the following two criteria: (1) protruding orange-red lesions visible on fundus examination; (2) characteristic polypoidal lesions evident (Figure 1) on ICGA. Patients with other neovascularized maculopathies, such as neovascular AMD, pathologic myopia, angioid streaks, presumed ocular histoplasmosis, and retinal angiomatous proliferation, were excluded. The characteristics of the study group are summarized in Table 1.

**Table 1:** Characteristics of the study population

**Demographic characteristics**	**PCV**	**Controls**	**p value**
Number of subjects	118	115	
Gender (male/female)	78/40	69/46	0.335
Mean age±SD (years)	65±8.4	69±8.7	<0.001
Age range (years)	43–85	50–87	

Abbreviations: PCV represents Polypoidal Choroidal Vasculopathy; SD represents standard deviation.

All control subjects underwent comprehensive ophthalmic examinations, and those with macular degeneration of any cause, macular changes (such as drusen or pigment abnormalities), or media opacities preventing clear visualization of the macula were excluded from the recruitment. All control subjects were unrelated to case subjects and were aged ≥50 years.

### Single nucleotide polymorphism selection

SNPs across *SERPING1*, including 5 kb upstream and downstream in the international haplotype map (HapMap) [29] for the Han Chinese in Beijing, China (CHB), were used to select tag SNPs. SNPs with a minor allele frequency above 5% were evaluated for linkage disequilibrium (LD) using Haploview software 4.1 [30]. A minimum threshold value of 0.8 for the r^2^ parameter was set in the Haploview software. r^2^ represents the multivariate coefficient of determination for all alleles that are to be captured. A previously reported SNP (rs2511989) was included as a tag SNP, using the forced inclusion option. The selected SNPs and SNPs captured by these SNPs are given in Table 2.

**Table 2:** Selected SNP and SNPS captured by these SNPS

**Selected SNP**	**SNPs captured by current SNP**
rs2509897	rs2511990 , rs2509897, rs2729376
rs1005510	rs1005510 , rs1005511, rs2511988
rs11603020	rs4926 , rs11229066, rs11229067, rs3824988, rs11603020, rs3758919
rs2511989	rs2508443 , rs2511989

Abbreviations: SNP represents single nucleotide polymorphism.

### Genotyping of participants

Peripheral blood sample were anticoagulated with ethylene diamine tetraacetic acid and stored at −80 °C before use. Genomic DNA from the blood by Nucleospin ®Blood XL kit (Macherey-Nagel GmbH & Co., KG Düren, Germany). The kit contains a silica membrane which can specifically bind DNA. After binding, DNA can be washed out by elute buffer. Genotyping was performed using PCR restriction fragment length polymorphism and direct sequencing.

The target DNA in *SERPING1* was amplified by PCR using relevant primers. The primers for each SNP were designed using Primer Premier 5.0 software (Premier Biosoft International, Palo Alto, CA). The primer sequences and restriction enzymes (New England Biolabs, Ipswich, MA) used for each SNP are presented in Table 3.

**Table 3:** Primers and restriction enzyme used in this study

**SNP**	**Primer sequence**	**AT (°C)**	**Enzyme**	**PCR product**	**Enzyme products**
rs2509897	F:TGGGAGCAGGTCTAGGATT	60.5	BslI (NEB)	170 bp	29 bp and 141 bp
	R: CAGGAAGAGCTTTAGTGAG				
rs1005510	F: GGCACAGTCCTCTAAAATAC	57.7	BbvI (NEB)	204 bp	102 bp and 102 bp
	R: CCTGACTATCCCTCATCTT				
rs11603020	F:TGGCAAAATGTGAGTCGTGTTCCT	63	BstNI (NEB)	293 bp	223 bp and 70 bp
	R: GCCAGTTGGGATCCTCTGGGC				
rs2511989	F: GAAGAAGGACTTTCAACTG	54.8	DdeI (NEB)	102 bp	21 bp and 81 bp
	R:TGAGAGGCAAATTCACTC				

Abbreviations: NEB represents New England Biolabs, Ipswich, MA; SNP represents single nucleotide polymorphism; AT represents annealing temperature.

Each PCR was performed in 20 μl of solution containing 10 μl of 2× Power Taq PCR MasterMix (Bioteke Corporation, Beijing, China), 8 pmol of primers, and 50 ng of genomic DNA. The cycling profile was: initial denaturation at 95 °C for 3 min, 33 cycles consisting of 95 °C for 30 s, proper annealing temperature (Table 3) for 30 s, 72 °C for 30 s, and a final extension for 10 min at 72 °C.

The digestion conditions contained 7.25 μl pure water, 1 μl NEB buffer, 0.25 μl enzyme and 1.5 μl PCR product for enzyme BslI, BbvI, and DdeI. The reaction conditions contained 7.15 μl pure water, 1 μl NEB buffer, 0.1 μl BSA, 0.25 μl enzyme and 1.5 μl PCR product for enzyme BstNI. The reaction temperature for enzyme BslI was 55 °C, for enzyme BstNI was 60 °C, and for enzyme BbvI and DdeI was 37 °C. After digestion, all fragments were resolved by electrophoresis on 8% polyacrylamide gel (PAG) at 30 W for 2 h. Subsequently, the PAG was silver stained [31]. Briefly, the gels were fixed with 1% nitric acid for 5 min and 10% alcohol for 3min. After several changes of water, the gels were incubated in 0.1% silver nitrate for 25 min. Then the gels were developed in 3% sodium carbonate and the reaction was terminated with acetic acid. To confirm the accuracy of the method used, randomly selected subjects (10% of all samples) were analyzed by direct sequencing (Shanghai Sangon Biologic Engineering Technology & Service Co. Ltd. Shanghai, China). All the primers used for direct sequencing are available on request.

### Statistical analysis

Age and gender differences between PCV and control subjects were assessed using the unpaired Student *t*-test and χ^2^ test, respectively, with SPSS 13.0 for windows software (SPSS Inc., Chicago, IL). Deviations from the Hardy–Weinberg equilibrium were tested using the exact test implemented in the software package PLINK, v1.06 [32]. Genotypes and allele frequencies between cases and controls were evaluated for each SNP using the chi-square test with PLINK. The odds ratio (OR) and corresponding 95% confidence interval (CI) were calculated relative to the minor allele. Haploview software was used to assess LD patterns and haplotype association statistics. Haplotype blocks were determined using the “4-gamete rule” option implemented in Haploview. A p value of <0.05 was considered statistically significant.

## Results

A total of 233 subjects were enrolled in this study, comprising 118 unrelated patients with PCV and 115 healthy control individuals (Table 1). The mean ages were 65±8.4 years for PCV patients and 69±8.7 years for healthy controls (p<0.001). The percentage of males was 66.1% in the PCV group and 60% in the control group (p=0.335).

**Figure 2:**
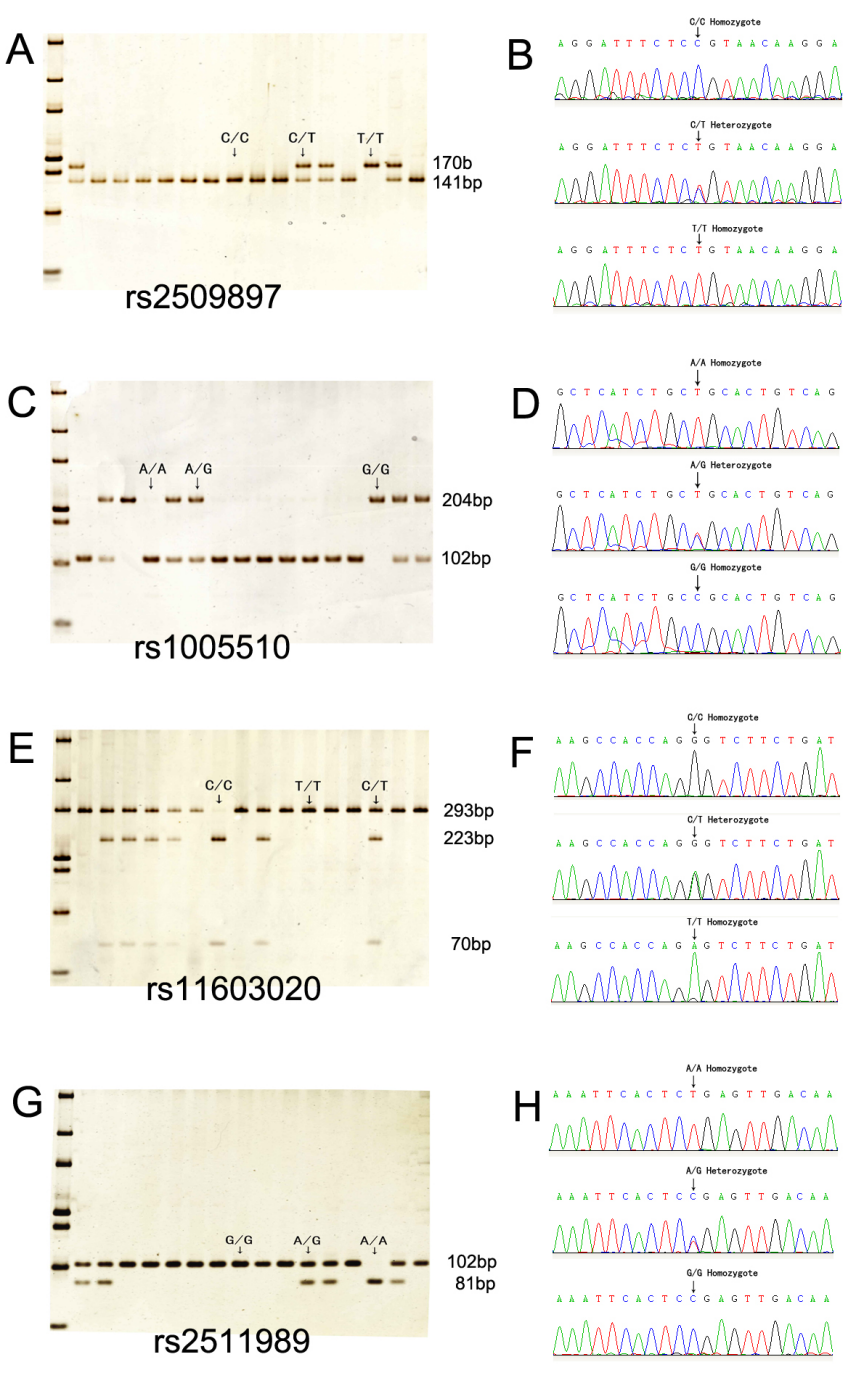
PCR restriction fragment length polymorphism (PCR-RFLP) and DNA sequencing for all tag single nucleotide polymorphisms (SNPs). **A**: Restriction analysis for rs2509897 resulted in digestible fragment (C/C), undigestible fragment (T/T), and heterozygote (C/T). **B**: Direct sequencing confirmed the restriction patterns for rs2509897. **C**: Restriction analysis for rs1005510 resulted in digestible fragment (A/A), undigestible fragment (G/G), and heterozygote (A/G). D. Reverse sequencing confirmed the restriction patterns for rs1005510. **E**: Restriction analysis for rs11603020 resulted in digestible fragment (C/C), undigestible fragment (T/T), and heterozygote (C/T). **F**: Reverse sequencing confirmed the restriction patterns for rs11603020. G. Restriction analysis for rs2511989 resulted in digestible fragment (A/A), undigestible fragment (G/G), and heterozygote (A/G). **H**: Reverse sequencing confirmed the restriction patterns for rs2511989.

Four SNPs were chosen as tag SNPs. Genotypes were determined successfully by restriction enzyme digestion in all subjects for the four SNPs and confirmed by direct sequencing (Figure 2). None of the four SNPs genotyped in this study showed significant deviation from Hardy–Weinberg equilibrium tests in both case and control subjects (all p>0.252).

The minor allele frequencies in each group for all tag SNPs genotyped and the results of the single-SNP association study are summarized in Table 4. The results of genotype frequencies for all tag SNPs are shown in Table 5. None of the four tag SNPs showed a significant association with PCV (Table 4 and Table 5). The OR for PCV in rs2511989 A/G heterozygotes compared with wild-type G/G homozygotes was 1.08 (95% CI 0.60–1.95). A similar comparison of the A/A homozygotes with the wild type yielded an OR of 3.04 (0.31–29.77).

**Table 4:** Association test for minor allele frequency between PCV and control subjects

**SNP**	**Position (bp)**	**Minor allele**	**PCV, n**	**Control, n**	**OR**	**95% CI**	**p value**
rs2509897	57119193	T	38 (0.161)	33 (0.144)	1.15	0.69–1.90	0.5984
rs1005510	57123798	G	61 (0.259)	52 (0.226)	1.19	0.78–1.83	0.4147
rs11603020	57130908	C	24 (0.102)	22 (0.096)	1.07	0.58–1.97	0.8269
rs2511989	57134901	A	37 (0.157)	31 (0.135)	1.19	0.71–2.00	0.5013

Abbreviations: CI represents confidence interval; OR represents odds ratio; PCV represents polypoidal choroidal vasculopathy; SNP represents single nucleotide polymorphism.

**Table 5:** Association test for genotype between PCV and control subjects

**SNP**	**Genotype**	**PCV, n (%)**	**Control, n (%)**	**OR**	**95%CI**	**P value**
rs2509897	CC	83 (70.3)	84 (73.0)			0.8543
	CT	32 (27.1)	29 (25.2)	1.12	0.70–2.01	
	TT	3 (2.5)	2 (1.7)	1.52	0.25–9.32	
rs1005510	AA	63 (53.4)	69 (60.0)			0.5643
	AG	49 (41.5)	40 (34.8)	1.34	0.78–2.30	
	GG	6 (5.1)	6 (5.2)	1.1	0.34–3.57	
rs11603020	TT	96 (81.4)	95 (82.6)			0.9647
	CT	20 (16.9)	18 (15.7)	1.1	0.55–2.21	
	CC	2 (1.7)	2 (1.7)	0.99	0.14–7.17	
rs2511989	GG	84 (71.2)	85 (73.9)			0.5963
	AG	31 (26.3)	29 (25.2)	1.08	0.60–1.95	
	AA	3 (2.5)	1 (0.9)	3.04	0.31–29.77	

Abbreviations: SNP represents single nucleotide polymorphism; PCV represents polypoidal choroidal vasculopathy; OR represents odds ratio; 95%CI represents 95% confidence intervals. OR and 95%CI were calculated to estimate risk size as comparison of the homozygote and heterozygote versus wild-type homozygote.

**Figure 3:**
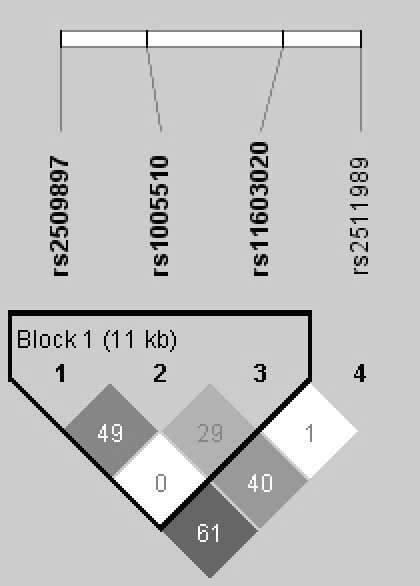
Linkage disequilibrium structure of serpin peptidase inhibitor, clade G, member 1 (*SERPING1)*. Linkage disequilibrium (LD) was measured using data from all subjects in this study. The haplotype blocks were determined using the “4-gamete rule” option implemented in the Haploview software. Each box provides estimated statistics of the coefficient of determination (r^2^), with darker shades representing stronger LD.

The pairwise LD structure was constructed with all SNPs genotyped (Figure 3). Three SNPs, rs2509897, rs1005510, and rs11603020, were placed within one haplotype block, and we examined haplotypes based on these three SNPs in the haplotype block. Details of the haplotypes and their frequencies in PCV and control subjects are presented in Table 6. No particular haplotype was found to be associated with a risk of PCV.

**Table 6:** Inferred haplotype frequencies and haplotype-based association study

Haplotype*	**PCV**	**Control**	**OR**	**95%CI**	**p value**
CAT	172.7 (0.732)	174.7 (0.759)	0.86	0.57-1.31	0.4911
TGT	35.1 (0.149)	30.1 (0.131)	1.16	0.69-1.96	0.5784
CGC	22.1 (0.094)	20.2 (0.088)	1.07	0.57-2.02	0.8198

All haplotypes with frequency >1% in the combined samples from PCV patients and controls are shown. *Haplotypes were defined by the following three contiguous SNPs: rs2509897, rs1005510, and rs11603020. Abbreviations: PCV represents polypoidal choroidal vasculopathy; OR represents odds ratio; 95%CI represents 95% confidence intervals.

## Discussion

In this study we investigated the association of *SERPING1* polymorphisms with PCV in a Chinese Han population. Our study showed that none of the four tag SNPs for *SERPING1* were associated with PCV. The genotyping methods used in this study were different from those in a previous study [26]. As shown previously [33,34], different genotyping technologies could yield inconsistent genotyping results. To validate the results of genotyping, two methods, PCR restriction fragment length polymorphism and direct sequencing, were used in this study.

PCV shares many similarities with neovascular AMD. However, the histopathological findings remain confusing [35], and there are distinct clinical differences between PCV and neovascular AMD, including morphologic features and disease progression [3], as well as response to therapy [36,37]. The clinical differences may indicate different genetic characteristics between PCV and neovascular AMD.

The complement system is a powerful component of innate immunity, which recognizes pathogens and unwanted host material and facilitates their elimination [38]. Activation of complement leading to membrane attack complex (MAC) formation can be triggered via three known pathways (the classical, lectin, and alternative pathways). The classical pathway is generally initiated by antibody interactions with foreign antigens. The protein encoded by *SERPING1* is an important complement regulator and plays a crucial part in suppressing the activity of C1. Inhibition of C1 prevents activation of C2 and C4, thus the cleavage of C3 into the anaphylatoxin C3a and the major fragment C3b is inhibited. C3b subsequently binds foreign structures and forms a complex with CFB that cleaves C5 (C3bBb, C5 convertase). This complex amplifies the complement response, resulting in the formation of MAC. Variants within several genes that code for proteins involved in the complement system are recognized to either significantly increase the risk of AMD (*CFH* [9-11] and *C3* [23]) or decrease the risk (*SERPING1* [26], *C2* and *CFB* [24]).

Due to the similarities between PCV and AMD, several studies were conducted to test the association between PCV and complement components. Previous studies [39,40] showed that several *CFH* variants (rs3753394, rs551397, rs800292, rs2274700, and rs1329428) but not the Y402H variant that is the most common variant in western AMD patients, were significantly associated with neovascular AMD in Chinese cohorts. A significant association between PCV and *CFH* variants (rs3753394 and rs800292) was observed in another Chinese cohort [18]. Thus it appeared that the alternative pathway might be implicated in the pathogenesis of both PCV and AMD.

Several studies showed a protective effect on neovascular AMD for *C2* and *BF* [24,41]. However, the association was not observed between PCV and *C2* in a Chinese cohort [18]. Ennis [26] reported a protective effect on AMD for *SERPING1*; however, no association between PCV and *SERPING1* was observed in this study. Both *C2* and *SERPING1* are crucial regulators of the classic complement pathway. Given the published data and the results of this study, we hypothesized that the classic pathway might play a minor role in the pathogenesis of PCV compared with neovascular AMD. However, further studies containing larger numbers of PCV and neovascular AMD patients should be conducted to test our hypotheses.

Drusen is abnormal accumulations of extracellular material that forms between the basal surface of the retinal pigmented epithelium and Bruch’s membrane. It is a significant risk factor for the development of AMD [42]. Previous studies showed that several complement components were found in drusen, including C3a and C5b-9 [43,44]. The prevalence of large soft drusen in the fellow eyes of unilateral PCV patients was much less than that among AMD patients [5,45]. This clinical difference between PCV and AMD can be partly explained by the genetic differences between them.

Potential limitations of our study should be mentioned. First, the PCV and control groups were not completely age matched. Second, we did not sample enough neovascular AMD patients and no comparison was conducted between PCV and neovascular AMD in this Chinese Han cohort. Third, no mechanistic and functional evaluations were conducted in this study.

In summary, this study has been conducted to investigate the association of *SERPING1* polymorphisms with PCV. To our knowledge, this issue has not been investigated to date. We found no evidence to support the role of any common *SERPING1* variation, including rs2511989, in the susceptibility to PCV in a Chinese Han population; thus the focus may be shifted to other loci in future PCV-related genetic association studies.
